# Can theory-driven implementation interventions help clinician champions promote opioid stewardship after childbirth? Protocol for a pragmatic implementation study

**DOI:** 10.3389/fgwh.2025.1504511

**Published:** 2025-03-14

**Authors:** Michelle H. Moniz, Amy M. Kilbourne, Alex F. Peahl, Jennifer F. Waljee, Shelytia Cocroft, Carey Simpson, Lisa Kane Low, Mark C. Bicket, Michael J. Englesbe, Molly J. Stout, Vidhya Gunaseelan, Althea Bourdeau, May Hu, Carrie Miller, Shawna N. Smith

**Affiliations:** ^1^ Department of Obstetrics and Gynecology, University of Michigan Medical School, Ann Arbor, MI, United States; ^2^ Institute for Healthcare Policy and Innovation, University of Michigan, Ann Arbor, MI, United States; ^3^ Obstetrics Initiative, Department of Obstetrics and Gynecology, University of Michigan, Ann Arbor, MI, United States; ^4^ Office of Research and Development, Veterans Health Administration, U.S. Department of Veterans Affairs, Washington, DC, United States; ^5^ Department of Learning Health Sciences, University of Michigan Medical School, Ann Arbor, MI, United States; ^6^ Department of Surgery, University of Michigan Medical School, Ann Arbor, MI, United States; ^7^ Opioid Prescribing Engagement Network, Ann Arbor, MI, United States; ^8^ Center for Healthcare Outcomes and Policy, University of Michigan, Ann Arbor, MI, United States; ^9^ School of Nursing, University of Michigan, Ann Arbor, MI, United States; ^10^ Department of Anesthesiology, University of Michigan Medical School, Ann Arbor, MI, United States; ^11^ Department of Plastic Surgery, University of Michigan Medical School, Ann Arbor, MI, United States; ^12^ Department of Health Management and Policy, University of Michigan School of Public Health, Ann Arbor, MI, United States; ^13^ Department of Psychiatry, University of Michigan Medical School, Ann Arbor, MI, United States

**Keywords:** acute pain, opioid, postpartum, implementation, guideline

## Abstract

**Background:**

Our objective is to determine the effect of a new national clinical practice guideline (CPG) for pain management after childbirth, as implemented with less vs. more intensive implementation support, on postpartum opioid prescribing.

**Methods:**

A quasi-experimental analysis will measure the impact of post-childbirth pain management guidelines on opioid prescribing in a statewide hospital collaborative, overall and among key patient subgroups at risk for inequitable care and outcomes. We will also use a randomized, non-responder design and mixed-methods approaches to evaluate the effects of Replicating Effective Programs (REP), a theory-driven, scalable implementation intervention, and Enhanced REP (E-REP; i.e., REP augmented with facilitation, which is individualized consultation with site champions to overcome local barriers) on the uptake of the CPG. The study will include hospitals within the Obstetrics Initiative (OBI), a perinatal collaborative quality initiative funded by Blue Cross Blue Shield of Michigan that includes 68 member hospitals serving more than 120,000 postpartum people, over approximately 15 months. Hospitals not initially responding to REP—defined by performance <15th percentile of all OBI hospitals for (a) inpatient order for opioid-sparing postpartum pain management (e.g., scheduled acetaminophen and non-steroidal anti-inflammatory drugs when not contraindicated), or (b) amount of opioid prescribed at discharge—will be allocated via block randomization to continue REP or to E-REP. Using interrupted time series analyses, the primary analysis will evaluate the rate of postpartum opioid-sparing prescribing metrics at the time of discharge (primary outcome) and opioid prescription refills and high-risk prescribing (secondary outcomes) before and after CPG implementation with REP. We will evaluate inequities in outcomes by patient, procedure, prescriber, and hospital factors. Exploratory analyses will examine temporal trends in patient-reported outcomes and the effects of continued REP vs. E-REP among slower-responder sites. We will evaluate implementation outcomes (e.g., acceptability, feasibility, costs, needed REP and E-REP adaptations) using clinician and patient surveys and qualitative methods (ClinicalTrials.gov identifier: NCT06285123).

**Discussion:**

Findings will inform refinements to the REP and E-REP interventions and add to the literature on the effectiveness of facilitation to promote uptake of evidence-based clinical practices in maternity care.

## 1 Introduction

Annually in the United States (U.S.), there are nearly four million birthing people who require pain management and comfort after childbirth. Postpartum opioid prescribing in the U.S. is widely variable at time of discharge from the childbirth hospitalization, putting individuals at risk of both insufficient pain management and the risks of opioid medications (1, 2). Inadequate pain management after childbirth can adversely affect maternal wellbeing, infant care and bonding, breastfeeding continuation, and risk of depression and chronic pain (3–7). Simultaneously, excess opioid prescribing confers risks of persistent opioid use and associated harms (8–12) and adverse health outcomes in infants (13). The peripartum period is also fraught with significant inequities in pain management experiences (14–20). In response, our team led a rigorous, multidisciplinary process to develop the Creating Optimal pain Management FOR Tailoring care (COMFORT) clinical practice guideline (CPG) for pain management after childbirth, which aims to mitigate peripartum opioid-related risks and existing inequities in pain management after childbirth.

When new clinical guidelines emerge, standard dissemination approaches, (i.e., publication and announcement) are often insufficient to change clinical practice. Intentional implementation efforts that focus on changing provider behavior and addressing organizational barriers such as local culture, leadership buy-in, and provider training and capacity are far more resource-intensive, but may more effectively encourage use of new CPGs.

*Replicating Effective Programs (REP)* is one theory-based (21–25) implementation intervention that has demonstrated effectiveness in accelerating clinical change compared to routine dissemination alone (23, 26). REP consists of four core implementation strategies: (1) User-friendly “packaging” of the CPG; (2) Structured provider training; (3) Performance feedback (e.g., reports with quarterly prescribing data); and 4) Brief technical assistance [e.g., group-based educational sessions for quality improvement (QI) champions]. REP advantageously requires no specialized implementation expertise, so it can be readily deployed by local implementers.

However, REP alone may be insufficient for ensuring effective adoption of the COMFORT CPG by all sites and uniform uptake in best practices by all clinicians within sites. In prior studies, REP-based approaches were effective in only 10%–15% of sites (27–36). To address heterogeneity of treatment effects, REP may be enhanced with facilitation [Enhanced REP (E-REP)] (37, 38). Facilitation is a process of interactive problem-solving within a supportive consultative relationship, with a goal of addressing local barriers and unanticipated implementation challenges and thereby enhancing clinician uptake of evidence-based practices (39–41). Facilitation is delivered by an expert who meets regularly with site QI (or other appropriate) leaders. In contrast to the brief, standardized technical assistance in REP, facilitation is intensive, individualized consultation that generates highly customized solutions that can potentially be sustained by local clinicians (37, 40, 42, 43). Moreover, E-REP may reveal the mechanisms that underlie differences in CPG implementation among marginalized patients, and potential strategies to close these gaps, given the opportunity to deeply engage with local partners. Though E-REP can be effective for sites not responding to REP alone (35, 44), facilitation is more resource-intensive and difficult to scale (45). Therefore, identifying healthcare settings most in need of facilitation is highly significant but has not been evaluated in maternity contexts to date (35, 46).

### 1.1 Primary aim 1

The primary aim of this study is to evaluate clinical outcomes before and after CPG implementation with REP. We hypothesize that REP initiation will be associated with greater reductions in rates and amounts of postpartum opioid prescribing at discharge from the childbirth hospitalization (primary outcome), via an increase in opioid-sparing pain management approaches.

### 1.2 Exploratory aim 1

To characterize temporal trends in patient-reported outcomes (i.e., pain intensity in the first week after childbirth, opioid consumption after discharge from the childbirth hospitalization, and satisfaction with pain management after discharge from the childbirth hospitalization). We hypothesize that opioid prescribing (primary outcome) will decrease without adverse effects on patient-reported outcomes.

### 1.3 Exploratory aim 2

To determine the effects of REP alone vs. REP with added facilitation (E-REP) among slower-responder sites. We hypothesize that, for slower-responders, adding facilitation (E-REP) is more effective in increasing opioid-sparing prescribing than continuing REP alone.

### 1.4 Exploratory aim 3

To evaluate for heterogeneity of treatment effects of REP and E-REP. Specifically, we will determine whether implementation intervention effectiveness is moderated by hospital-level factors (e.g., early positive change in COMFORT practices, perceived hospital leadership support for COMFORT adoption). Results of these analyses may be used to construct a more deeply tailored adaptive intervention that further improves uptake of the COMFORT CPG.

### 1.5 Exploratory aim 4

To describe CPG implementation using REP and E-REP at the site level, including feasibility and acceptability to patients and providers, needed REP and E-REP adaptations, and costs of these implementation interventions.

The result of this study will be an optimized, potentially adaptive implementation intervention that could inform QI efforts in maternity units—and other acute pain settings—to improve pain management, outcomes, and outcome inequities.

## 2 Methods and analysis

### 2.1 Study design and duration

**Figure 1:**
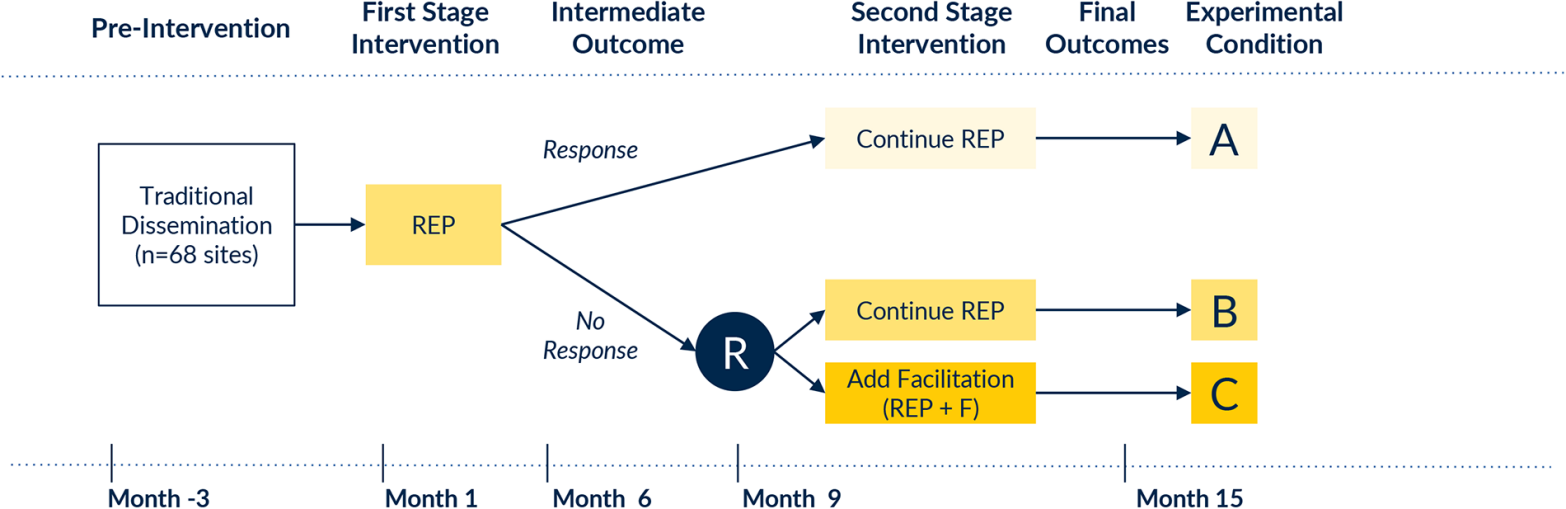
Study design. R, randomization; REP, Replicating Effective Programs; E-REP, Enhanced Replicating Effective Programs.

We will use a quasi-experimental design to evaluate temporal trends in clinical outcomes before and after CPG implementation with REP. A non-responder randomized trial (47, 48) will also be conducted subsequently to test the effects of REP and E-REP on clinical outcomes—overall and among key subgroups. In addition to clinical outcomes, we will also measure implementation outcomes, (e.g., adoption, acceptability, feasibility, and appropriateness of REP and E-REP; needed REP/E-REP adaptations; costs of delivering E-REP) to guide REP and E-REP enhancements. The unit of randomization will be the hospital. Figure 1 outlines the study design and timeline, resulting in a group of responder sites that continue to receive REP, a slower-responder arm that receives only REP, and a slower-responder arm that receives E-REP. It is anticipated that the study will take approximately 15 months to complete. Because our QI interventions involve provider education and consultation in use of guidelines that meet or exceed standards of practice, this study was deemed exempt in its use of survey and qualitative data collection and secondary analysis of existing data by the University of Michigan institutional review board (HUM00248235 and HUM00248331). Our study is reported according to SPIRIT guidelines.

### 2.2 Study setting and population

The study setting is the Obstetrics Initiative (OBI), a Blue Cross Blue Shield of Michigan (BCBSM)-funded collaborative of Michigan hospitals dedicated to maternity care quality improvement. OBI's 68 member hospitals constitute diverse maternity practice settings ranging from urban to rural, academic to community, and high- to low-volume maternity units. Each site has a designated QI champion team, including at least one physician champion, one nurse/midwife champion, and one clinical data abstractor. Each hospital's clinical data abstractor reviews hospital medical record data and enters initiative-specific data into an OBI clinical registry workstation. OBI hospitals receive site-specific and collaborative-wide data on a variety of maternity quality indicators. Site champions attend OBI's twice annual in-person collaborative-wide meetings, as well as virtual sessions throughout the year for education, best practice sharing, and peer mentorship. Finally, OBI sites receive Pay for Performance (P4P) points for participation in OBI activities and quality metrics obtained, with associated payments to OBI hospitals through BCBSM's Value-Based Partnerships program.

In January 2024, OBI launched a new QI initiative, “Bringing Our Patients COMFORT,” to promote adoption of the COMFORT CPG across member hospitals. To support this initiative, all OBI hospitals are eligible for P4P points for COMFORT CPG-concordant clinical behaviors—specifically, ordering scheduled acetaminophen and scheduled oral non-steroidal anti-inflammatory drugs (NSAIDs) for eligible patients and discharge opioid prescribing within CPG-concordant ranges. All hospitals will receive REP- or E-REP-based support and tools from the OBI Coordinating Center. To the degree that OBI clinical champions share these resources on their units, maternity clinicians staffing OBI hospitals will be exposed to these activities. To the degree that OBI hospitals adopt the COMFORT CPG, birthing populations at these sites will be exposed to guideline-concordant care.

### 2.3 Evidence-based practice

**Figure 2:**
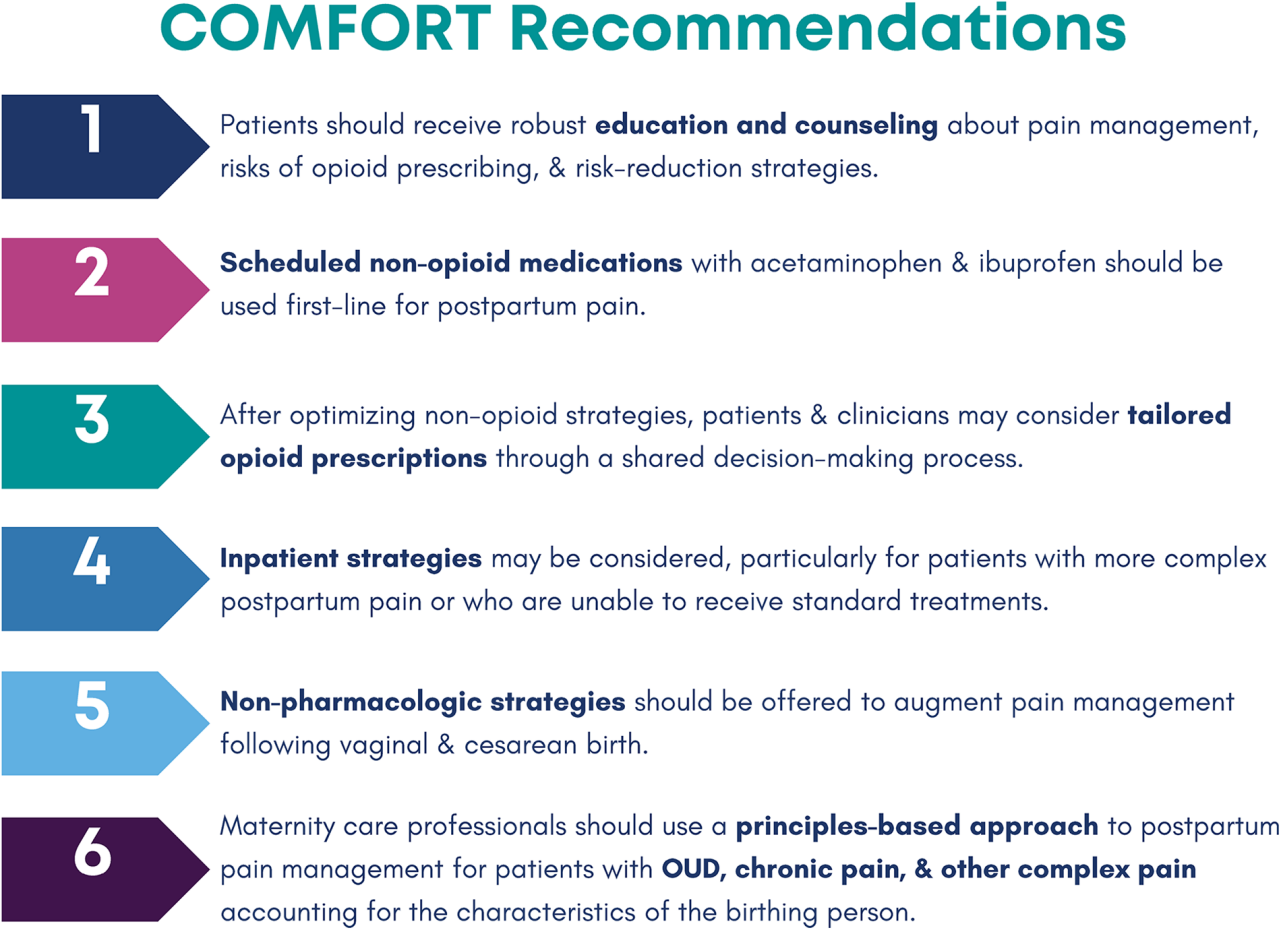
Key components of the COMFORT CPG. CPG, clinical practice guidelines; OUD, opioid use disorder.

The COMFORT CPG was created using the RAND/UCLA Appropriateness Method (49), a rigorous, modified eDelphi process that incorporates published evidence (collated via a systematic review) and expert opinion to arrive at clinical recommendations. The COMFORT CPG development process explicitly involved public members and considered health equity in all stages of CPG development, informed by best practices collated by the GRADE Working Group (50–53) and a recent scoping review (54). Key components of the COMFORT CPG are highlighted in Figure 2.

### 2.4 Implementation interventions

We designed REP and E-REP implementation interventions informed by prior qualitative work involving individual semi-structured interviews with 31 patients and 22 clinicians and six focus groups with 43 maternity care QI champions (55). We specify the REP and E-REP interventions briefly in Table 1, and fully elsewhere (55).

**Table 1:** Brief implementation intervention specification.

Intervention	Core components	Tools	Target
P4P	Payer provides additional payment, once annually, for CPG-concordant performance in prior year	• OBI P4P metric focused on opioid-sparing management and opioid prescribing	Hospital
REP	1. User-friendly packaging of CPG 2. Structured training of unit-based QI teams and clinicians in COMFORT CPG 3. Brief technical assistance (website with resources and OBI Coordinating Center staff available to address questions) 4. Performance feedback on prescribing behaviors at the hospital level, compared to peers in OBI	• Clinician practice guide • Sample unit protocol • COMFORT CPG webinar recording • Champion roles/responsibilities • C-suite letter • Unit assessment tool • Strategies checklist • QI blueprint • Frequently Asked Questions document • Protocol template • Electronic health record template • Patient educational materials	Hospital QI champion team
Facilitation	Trained facilitator meets with unit-based teams responsible for implementing COMFORT CPG to engage in: 1. Building supportive relationships 2. Problem-solving activities 3. Ongoing monitoring 4. Planning for sustainment	• Facilitation manual that guides the facilitator in active discussions with QI teams	Hospital QI champion team

P4P, pay for performance; CPG, clinical practice guideline; OBI, Obstetrics Initiative; QI, quality improvement; COMFORT, Creating Optimal pain Management FOR Tailoring care.

### 2.5 Trial procedures

Pre-intervention (Month -3 to 0): All OBI hospital champions are notified about the COMFORT CPG using standard dissemination techniques (e.g., email, announcements at collaborative-wide meetings, postings on collaborative website). First-stage intervention (Month 1–9): All hospitals receive the REP implementation intervention, including: a web-based toolkit with the CPG and related implementation resources; structured clinician training offered to all maternity clinicians through a virtual educational session with an asynchronous viewing option; performance feedback about opioid prescribing and other key outcome metrics; and peer mentorship opportunities (e.g., didactic content at OBI's in-person meetings). Determination of non-response: Hospital response to REP is assessed at Month 9 (at which time Month 6 OBI registry data will be available for analysis). Performance in Months 1–6 will be used to determine responder status. Response will be defined based on hospital concordance with specific CPG recommendations; specifically: a) provision of opioid-sparing pain management during the childbirth hospitalization, and b) amount of opioid prescribing at time of discharge. OBI hospitals with the highest 15th percentile performance on both indicators (estimated *n* = 10 sites) will be considered “Rapid Responder” sites. The remaining 58 sites will be deemed “Slower-Responders,” enabling capture of the part of the performance distribution that is least compliant with CPG recommendations. We designated these *a priori* counts of hospitals a) based on prior work showing about 15% of sites respond to REP alone (27–36), and b) due to OBI Coordinating Center capacity to offer facilitation to up to 28 sites concurrently. If needed, we will modify these thresholds based on observed comparative performance of OBI sites. Second-stage intervention for slower-responders (Month 10–15): Hospitals that respond to REP will continue it. Hospitals that do not respond will be randomized 1:1 by the study data analyst to continue REP (as some hospitals simply require more time to respond) or to E-REP. Randomization will be stratified by rural vs. urban status, delivery volume (above vs. below 1,500 births annually), and site-average rate of opioid-sparing interventions in Months 4–6 to ensure that intervention groups are balanced for site variables that may highly correlate with outcomes. The study analyst will generate stratified permuted-block random allocation lists (block sizes of 2, 4, and 6) using a computer program such as PROC PLAN in SAS. A site will be considered randomized once the study analyst informs the study coordinator of each site's random assignment. For hospitals randomized to E-REP, facilitation will be based on the COMFORT facilitation guide and delivered by a trained facilitator, with support from OBI Coordinating Center Outreach and Engagement Coordinators, who have strong relationships with OBI member hospitals. The facilitator will offer to meet (virtually, or in person at the site's request) with the site's QI team to review performance trends, identify key barriers and facilitators to improvement, select improvement strategies to execute, and specify improvement targets. The facilitator will then offer recurring virtual meetings with site QI teams to promote CPG uptake, with meeting cadence adjusted based on site needs and preferences.

### 2.6 Ensuring fidelity to REP and E-REP

Fidelity monitoring will be used to assess whether each site is receiving the core components of each implementation intervention (REP and E-REP). Fidelity to REP will be defined based on study team documentation of dates when trainings are offered and materials are uploaded to the OBI website, and ascertained via routine OBI administrative data. Fidelity to E-REP will be defined using the following criteria: (a) number of sessions offered and number of sessions completed by the facilitator with champions at each site; (b) number of documented barriers, facilitators, and specific goals to enhance CPG uptake; (c) documentation of site champion team strengths and available opportunities to influence site activities and overcome barriers; and (d) number of facilitation core components delivered [using tracking tools deployed in other large-scale implementation trials (56) and adapted for this study]. Data to determine E-REP fidelity will come from facilitation meeting logs completed by study staff. Throughout the trial, the study team will also collect data on adaptations made to REP and E-REP using the FRAME and FRAME-IS tools (57, 58).

### 2.7 Data sources

OBI has access to multiple data streams including a clinical registry, claims data from BCBSM and Medicaid plans (representing over 75% of market share in Michigan), and prescription drug monitoring program data with an approximately 90-day lag from the index procedure (59). The OBI clinical registry includes a subset of births (approximately 25,000 individuals in the nulliparous, term, singleton, vertex population are abstracted annually), and trial outcome measures will occur in this registry cohort as a signal of the unit's practices across its entire birthing population. Eligible births are abstracted to the OBI registry within 90 days by on-site clinical data abstractors at each OBI hospital. Our analytic sample will exclude those with opioid use disorder and complex pain, as these populations have different pain management needs than the broader birthing population.

Variables are collected and attributed to the hospital level to enable outcome adjustment for patient complexity (e.g., age, race and ethnicity, chronic conditions); procedural factors (e.g., mode of delivery); hospital characteristics (e.g., annual delivery volume, payer mix); and provider characteristics (e.g., specialty) (Table 2).

**Table 2:** Collaborative data and covariates in the analysis.

Variable	Attributes
Patient-level factors	
Demographic attributes	Age, race-ethnicity, insurance type, and ZIP code (rural-urban status; neighborhood social vulnerability).
Clinical attributes	Medical and mental health comorbid conditions.
Preoperative medication use	Controlled substances, including opioids, benzodiazepines, sedatives, and anxiolytic prescription fills prior to childbirth.
Procedure-level factors	
Type	(a) vaginal birth without laceration, (b) vaginal birth with laceration, (c) cesarean birth.
Urgency	Planned vs. unplanned cesarean.
Complications	Severe maternal morbidity.
Hospital-level factors	
Hospital attributes	Region, number of beds, birth volume, profit status, teaching status, payer mix, disproportionate share index, and urbanicity.
Prescriber-level factors	
Provider attributes	Specialty (Obstetrician, Family Physician, Nurse Midwife, Other).

Collaborative registry (drawn from the electronic health record), linked to prescription drug monitoring program data.

### 2.8 Effectiveness outcomes

We will measure CPG effectiveness as implemented by REP or E-REP using prescribing outcomes and patient-reported outcomes (Table 3). Our primary outcomes include: (1) the proportion of patients receiving an opioid prescription at discharge; and (2) the median opioid prescription size at discharge reported in oral morphine equivalents (OMEs)—overall, by hospital, and among key subgroups. Secondary outcomes include refilled prescriptions (i.e., additional opioid prescriptions filled within 30 days of discharge after childbirth) and high-risk prescribing (i.e., prescription >50 OMEs/day, overlapping benzodiazepine and opioid prescriptions, or overlapping opioid prescriptions—all within 30 days of discharge after childbirth), which may elevate risk of opioid-related harms (60–62). Exploratory effectiveness outcomes include patient-reported pain intensity in the first week after childbirth, opioid consumption after discharge from the childbirth hospitalization, and satisfaction with pain management after discharge from the childbirth hospitalization—all captured by validated survey measures on surveys sent to eligible patients abstracted into the OBI registry (63, 64). We will examine all effectiveness outcomes overall and by procedure type (e.g., vaginal births without additional procedures, cesarean births).

**Table 3:** Measures of CPG effectiveness as implemented with REP or E-REP.

Outcome	Measure	Data source
Primary	Rate of opioid prescribing at discharge from childbirth hospitalization.	Registry
	Amount of opioid prescribing at discharge from childbirth hospitalization.	
Secondary	Rate of refill opioid prescriptions within 30 days of discharge from childbirth hospitalization.	Registry-prescription drug monitoring program
	High-risk prescribing within 3 days of discharge from childbirth hospitalization (e.g., prescription size >50 OME, overlapping opioid benzodiazepine prescription, overlapping opioid prescriptions).	
Exploratory	Pain intensity (PEG score) in first week after childbirth.	Registry-patient voices
	Opioid consumption after discharge from childbirth hospitalization.	
	Satisfaction with pain management after discharge from childbirth hospitalization.	

OME, oral morphine equivalent; PEG, Pain, Enjoyment, General activities.

### 2.9 Primary analysis of the association between REP and effectiveness outcomes

Interrupted time series analysis will compare monthly rates of each outcome (e.g., opioid prescribing rate and amount) at the hospital level, before and after CPG implementation with REP. This quasi-experimental approach allows us to estimate the causal effect of the CPG as implemented with REP and identify the attributes of hospitals that tend to respond to REP alone. All analyses will be performed at the hospital level in an intent-to-treat manner among all OBI hospitals (groups A, B, and C in Figure 1), regardless of fidelity to REP and E-REP. We will use aggregated outcomes within each procedure type in our primary analysis. Time series plots will be used to visually inspect the effect of the intervention and presence of trends, cyclical patterns, and outliers. Serial autocorrelation, non-stationarity, and seasonality will be assessed using Cumby-Huizinga tests, Dickey-Fuller unit root test, and visual inspection of residual plots. If autocorrelation is present, we can employ robust Newey-West errors with the appropriate number of lags.

#### 2.9.1 Extensions of the analysis

We will construct mixed-effects logistic models for each effectiveness outcome (Table 3), incorporating time to detect trend and all covariates in Table 2 to explore heterogeneity in response to REP. The clustering effects of patients cared for by the same provider within a hospital will be accounted for to allow for REP's effects to vary at the provider/hospital level.

#### 2.9.2 Sample size and power calculation

In our preliminary analyses, OBI's average hospital rate of opioid prescribing at discharge among pregnant individuals was estimated to be 26% and hospital-level intraclass correlation coefficient (ICC) regarding the prescribing patterns was 0.25. We conservatively estimate 68 hospitals participating in OBI throughout the study period, each with an average of 100 deliveries in three months. We have sufficient power (0.823) to detect a decline as small as 2% (from 26% to 24%) in the opioid prescribing rate. Based on these estimates, we have >0.81 power to detect even small declines in opioid prescribing across 68 hospitals that receive the COMFORT CPG implemented with REP. When stratified by mode of delivery, we estimated a power of 0.864 to detect a 3% reduction in prescribing rate (from 88% to 85%) among cesarean births and a power of 0.843 to detect a 1.2% reduction in prescribing rate (from 6% to 4.8%) among vaginal births.

### 2.10 Exploratory analysis of the effects of REP vs. E-REP among slower-responders

While our primary analysis uses an observational design to evaluate the CPG as implemented by REP, an immediately publicly available and scalable implementation intervention, our exploratory analyses will identify how best to remediate sites that do not rapidly respond to the CPG as implemented by REP alone. Analyses will include all registry patients undergoing childbirth at the randomized slower-responder hospitals during the second intervention stage and compare effectiveness outcomes after exposure to the CPG implemented by REP vs. E-REP. Mixed-effect logistic regression models will be used to analyze each effectiveness outcome, accounting for clustering of individuals within hospitals (i.e., a 3-level model that includes fixed effects for the intercept, provider, and site-specific baseline opioid prescribing rate). A random effect will be included for provider and site. We will cluster standard errors in a way that accounts for clustering of patients within providers within hospitals.

Exploratory Aim 3 analyses will assess whether the implementation intervention effectiveness is moderated by baseline or time-varying hospital factors, to capitalize on hospital-level heterogeneity to inform the potentially adaptive implementation intervention. From prior literature, we have identified several candidate variables for testing moderation of the effects of REP vs. E-REP. Specifically, we will assess whether implementation intervention effectiveness is moderated by early positive change in COMFORT practices (observed in trial months 1–6); higher proportion of attendings who are private practice physicians; perceived hospital administrator support for adoption of COMFORT (as reported by the QI team leading COMFORT implementation); and number of hospital barriers to COMFORT (as reported at first measurement post-randomization). Among rapid responders, we hypothesize that few, if any, hospitals will demonstrate worsening performance after determination of response status, so we do not have pre-specified moderator analyses for this group.

#### 2.10.1 Power

An estimated 58 hospitals will be deemed slower-responders and randomized to either continue REP or to E-REP. With at least 100 patients per site, a mixed-effects logistic regression, provider-level ICC of 0.05, hospital-level ICC of 0.20, and two-sided alpha = 0.05, we have 85% power to detect an 8% change in compliance for opioid sparing prescribing, from 82% to 90%, by Month 6 post-randomization—a smaller difference than in our prior pilot work (65) that improved adherence from 38% to 61%. Among cesarean births, we estimated 0.838 power in detecting a 10% increase in compliance (from 80% to 90%), and among vaginal births, a power of 0.806 in detecting an 8% increase (from 82% to 90%).

### 2.11 Evaluation of implementation

We selected outcomes using the RE-AIM framework, which includes a comprehensive set of theory-based outcomes to evaluate implementation interventions (Table 4) (66). We will assess Reach, the number and proportion of patients receiving CPG-concordant care (defined by our primary analysis of effectiveness, examined pre/post CPG implementation, and our exploratory slower-responder analysis, examined post-implementation among slower-responder sites receiving REP vs. EREP), including by historically marginalized groups (e.g., populations marginalized by race or ethnicity), with a goal of identifying inequities in care delivery. Adoption metrics will characterize variation in hospital and provider uptake of CPGs and their attributes. Implementation outcomes: We will examine providers’ perceived feasibility, acceptability, and appropriateness of REP and E-REP (via validated measures on provider surveys and qualitative methods) (67, 68). The study team will also estimate the OBI Coordinating Center costs of creating REP and delivering E-REP facilitation using time-driven, activity-based costing methods (69, 70). We will estimate maintenance outcomes (for effectiveness, adoption, and reach metrics) at Month 24.

**Table 4:** Implementation outcomes by RE-AIM domain.

Domain	Source	Months collected
Reach	Registry	−4 to 18
Number, percent, and characteristics of patients receiving CPG-concordant care, overall and by subgroup to detect inequities		
Effectiveness	Registry	1–18
Prescribing and patient-reported measures (Table 2)		
Adoption	Registry	1–18
Number, percent, and characteristics of responding hospitals vs. all hospitals		
Number, percent, and characteristics of providers ordering CPG-concordant pain management interventions in at least 90% of procedures vs. all providers		
Implementation		
Perceived feasibility, acceptability, and appropriateness of REP and E-REP using surveys and qualitative methods	Study team	15–24
Cost of each implementation intervention (REP vs. E-REP)	Study team	1–18
Maintenance	Registry	19–24
6-month follow-up on all effectiveness outcomes		
Qualitative Implementation Outcomes	Patient and provider data	19–24
Assessment of mechanisms of action, factors affecting implementation, and local adaptations to CPGs		

CPG, clinical practice guideline; REP, replicating effective programs; E-REP, enhanced REP.

Finally, we will conduct robust qualitative work to elucidate the mechanisms underlying observed response to REP and E-REP and effects of the CPG. We will purposively sample eight hospitals (one high-performer and one low-performer hospital from Group A and one high- and two low-performer hospitals from Group B and Group C in Figure 1, with performance defined by our primary effectiveness analyses). We will invite site champions and up to five designated key informants from these hospitals to participate in semi-structured interviews or focus groups. The diversity in OBI practice settings will allow for maximum flexibility in sampling of facilities based on CPG implementation performance and by any organizational determinants noted to be significant drivers of implementation. We will also conduct semi-structured interviews with patients (*n* = 3 per site), with participants purposively sampled from consenting patient-reported outcome survey respondents.

## 3 Discussion

This study will provide the first prospective evaluation of the effectiveness of the COMFORT CPG on postpartum pain management in a statewide collaborative quality initiative. To date, this is one of the only prospective studies to design a potentially adaptive site-level implementation intervention (REP) in real-world maternity settings in the U.S. This study is also one of the first to test the augmentation of REP with facilitation (E-REP) among maternity units that exhibit slower response after six months, as well as one of the first large-scale, prospective evaluations of implementation interventions designed to promote opioid stewardship within collaborative quality initiatives. This study will elucidate how best to adapt REP to the unique context of maternity care and whether augmentation of REP is needed, or whether in some circumstances, withholding augmentation may result in delayed implementation effect of REP alone among slower-responder maternity units.

Improving the quality of maternity care is a national priority in the U.S. and many other countries, but there is a paucity of rigorous data on effective maternity care QI interventions. There have been few rigorous trials of implementation interventions in real-world maternity settings (71). A recent systematic review including 15 large-scale improvement programs focused on intrapartum care in the English National Health Service identified weak or absent evaluation in most studies, suggesting an urgent need to improve the evaluation of improvement initiatives in order to optimize their impact and ensure accountability for how improvement resources are utilized and how lessons learned are aggregated to improve clinical outcomes (72). Without sound evaluation, resources may be spent on ineffective QI interventions, misdirected to sites that do not need additional support, or geared toward interventions or implementation strategies that do not benefit the most at-risk, marginalized populations and that perpetuate inequities in maternity care and outcomes.

REP has documented success in real-world healthcare settings, but QI leaders may face organizational barriers (e.g., lack of leadership buy-in, competing priorities, provider resistance) that are beyond the scope of REP's key components (i.e., QI toolkits, structured clinician training, performance feedback, and group mentorship). Addressing these complex barriers may require more sustained strategic thinking, problem-solving, and organizational alliances. Facilitation may provide the interpersonal support that equips and motivates site champions to generate highly customized, local solutions to these barriers—and, ultimately, more effective CPG implementation. Facilitation may also uncover barriers in the effectiveness and implementation of CPGs across certain groups of hospitals, providers, or patients and may support more focused strategies to close these gaps and achieve equity in care delivery. This study will help determine the value of REP and E-REP and their effects on COMFORT CPG uptake in maternity units and patient outcomes.

While this study has numerous strengths, including the use of a novel implementation study design within a real-world perinatal quality collaborative, there are limitations that should be noted. Planned facilitation fidelity measures do not include direct observations of sessions to support implementation at the site level due to resource constraints. There is also a chance of contamination between REP and E-REP, because OBI sites have the option to request additional support from Coordinating Center staff as needed. This risk is unlikely, as site-initiated requests are rare. In addition, while we have robust mechanisms to capture opioid prescribing, opioid consumption, and pain management, our capture of non-prescribed analgesic use (opioids/NSAIDS/acetaminophen) post-discharge is limited. Finally, although OBI hospitals represent a range of practice settings and patient populations served, they may not be representative of all practices and populations nationwide.

Nearly 4 million individuals undergo childbirth annually in the U.S. The COMFORT CPG—if effectively implemented—holds promise to reduce variation in postpartum pain management practices, reduce opioid risks, and promote more equitable care and outcomes. The results of this study will inform implementation of the COMFORT CPG in Michigan, as well as how to conduct practical implementation studies in perinatal quality collaboratives. This study will also enable those leading perinatal QI initiatives to determine the potential added value of more intensive implementation strategies and to tailor QI resource allocation across sites that vary in need of support for adoption of evidence-based maternity practices.
